# Dietary patterns related to total mortality and cancer mortality in the United States

**DOI:** 10.1007/s10552-021-01478-2

**Published:** 2021-08-11

**Authors:** Marcela R. Entwistle, Donald Schweizer, Ricardo Cisneros

**Affiliations:** 1 Department of Public Health, College of Social Sciences, Humanities and Arts, University of California, 5200 North Lake Road, Merced, CA 95343 USA; 2 Pacific Southwest Region, USDA Forest Service, Clovis, CA USA

**Keywords:** Dietary patterns, United States, Total mortality, Cancer mortality

## Abstract

**Purpose:**

This study investigated the association between dietary patterns, total mortality, and cancer mortality in the United States.

**Methods:**

We identified the four major dietary patterns at baseline from 13,466 participants of the NHANES III cohort using principal component analysis (PCA). Dietary patterns were categorized into ‘prudent’ (fruits and vegetables), ‘western’ (red meat, sweets, pastries, oils), ‘traditional’ (red meat, legumes, potatoes**,** bread), and ‘fish and alcohol’. We estimated hazard ratios for total mortality, and cancer mortality using Cox regression models.

**Results:**

A total of 4,963 deaths were documented after a mean follow-up of 19.59 years. Higher adherence to the ‘prudent’ pattern was associated with the lowest risk of total mortality (5th vs. 1st quintile HR 0.90, 95% CI 0.82–0.98), with evidence that all-cause mortality decreased as consumption of the pattern increased. No evidence was found that the ‘prudent’ pattern reduced cancer mortality. The ‘western’ and the ‘traditional’ patterns were associated with up to 22% and 16% increased risk for total mortality (5th vs. 1st quintile HR 1.22, 95% CI 1.11–1.34; and 5th vs. 1st quintile HR 1.16, 95% CI 1.06–1.27, respectively), and up to 33% and 15% increased risk for cancer mortality (5th vs. 1st quintile HR 1.33, 95% CI 1.10–1.62; and 5th vs. 1st quintile HR 1.15, 95% CI 1.06–1.24, respectively). The associations between adherence to the ‘fish and alcohol’ pattern and total mortality, and cancer mortality were not statistically significant.

**Conclusion:**

Higher adherence to the ‘prudent’ diet decreased the risk of all-cause mortality but did not affect cancer mortality. Greater adherence to the ‘western’ and ‘traditional’ diet increased the risk of total mortality and mortality due to cancer.

## Introduction

In the last decades, efforts have been made to quantify the burden of disease attributable to specific dietary factors [1–3]. The study of diet and disease associations has traditionally originated from the consumption of single nutrients or foods. Dietary patterns describe diet quality and explain the total variability in food intake [4, 5]. Additionally, dietary patterns account for complex interactions that occur among foods and nutrients [6]. Therefore, the study of dietary patterns provides a clearer view of food and nutrient consumption and could better predict long-term outcomes such as mortality due to dietary exposure [7].

Several studies evaluated the effect of dietary patterns and mortality in other countries [8–11]. In the United States, studies have shown the association between adherence to specific dietary patterns and chronic diseases, including obesity [12, 13], cardiovascular disease, and diabetes [14]. Moreover, only a few studies have investigated the relationship between specific dietary patterns and total mortality [15–20]. Most of these studies have focused on particular race and ethnicities [17], older adults [15, 20], or participants with specific health conditions, such as chronic kidney disease [19]. Therefore, evidence of the association between adherence to dietary patterns and total mortality in the United States in the general population is still limited.

This study evaluates the association between dietary patterns, total mortality, and cancer mortality in the NHANES III adult cohort in the U.S. We hypothesize that dietary patterns characterized by consumption of nutrient-rich foods are associated with lower total mortality, and mortality due to cancer in the United States. Conversely, we hypothesize that dietary patterns that reflect poor nutrition are associated with higher total mortality and mortality due to cancer. We used data from 13,466 adults from the National Health and Nutrition Examination Survey III (NHANES III). We identified the predominant dietary patterns in relationship with total mortality and mortality due to cancer.

## Materials and methods

### Study population

The study used data from the NHANES III, a nationally representative sample of the civilian, non-institutionalized U.S. population conducted between 1988 and 1994 by the National Center for Health Statistics (NCHS) of the Centers for Disease Control and Prevention (CDC). The NHANES study protocol obtained ethical approval from the CDC/NCHS Ethics Review Board and received informed consent from all participants. The current study included 13,466 participants 18 to 90 years old, with complete data on mortality status, diet, and relevant covariates. The examination component involved examinations by a health professional, including physicians, dentists, and health technicians. Detailed information on the standardized protocols used in NHANES III has been previously published [21].

### Assessment of dietary exposure

During the interviews, dietary records providing detailed information about the foods and beverages were collected using computer-assisted software. We followed the recommendation from the NHANES III dietary methodology committee to use the 24-h recall as the principal methodology to provide detailed quantitative food and nutrient intake assessment, and the food frequency questionnaire (FFQ) to supplement data from the 24-h recall to provide typical or qualitative data [22, 23]. Therefore, the percentage of daily calories and macronutrients was calculated based on the information gathered during the 24-h food recall interview, and the usual diet assessment was based on the FFQ that included 60 food items and beverages. This was calculated in standardized portion sizes and nutrient intakes as grams per day. Participants without complete dietary data and those that reported excessively high or low values for total food or energy intake (less than 600 kcal/day in or more than 4,200 kcal/day) were excluded based on previously defined cut-offs [24–26]. Food groups were classified based on their nutrient profiles based according to the USDA Nutrient Lists from Standard Reference Legacy [27]. The food groups and items are shown in Table 1.

**Table 1:** Food groups and food items

Food groups	Food items
Dairy	Chocolate milk, milk, yogurt, beverages made with milk and creams, cheese, and cheese dishes
Red meat	Beef and pork
Poultry	Poultry
Processed meat	Processed meat, entrails
Fish	Fish and seafood
Eggs	Eggs and egg products
Vegetables	Soups and dishes with vegetables, all types of vegetables
Fruit	Fruits and fruit juices
Legumes	Bean including kidney, pinto and black beans, lentils, chickpeas, and rice
Potatoes	All types of potatoes, including sweet potatoes and yams
Nuts and seeds	Nuts and seeds
Cereal	Bran, fiber, cold and hot cereals, box cereals
Bread	bread, tortillas, and past
Sweets and pastries	pastries, chocolate, candy
Caffeinated beverages	Tea and coffee
Carbonated drinks	Regular and diet sodas, colas, drinks with vitamin C, beer
Alcohol beverages	wine, champagne, hard liquors
Oils	Butter, Margarine, vegetable oils and salad dressings

### Assessment of covariates

The sociodemographic and lifestyle characteristics information was collected during the face-to-face household interview. The participants’ characteristics included in the analysis were: age (years at the time of the recruitment); sex (male or female); educational attainment (years of schooling completed); and current smoking status (yes, no, or missed to answer). The medical history of chronic diseases, including ischemic heart disease, stroke, hypertension, diabetes, was also collected during the household interview. The variables were dichotomized as yes or no. The questions participants were asked were: *“Has a doctor ever told you that you had a heart attack?”; “Has a doctor ever told you that you had a stroke?”; “Have you ever been told by a doctor or other health professional that you had hypertension, also called high blood pressure?”; “Have you ever been told by a doctor that you have diabetes?”; “Has a doctor ever told you that you had skin cancer?”* and *“Has a doctor ever told you that you had any other cancer?”.* The collection of anthropometric measurements during the physical examination included standing height and weight using standard protocols. We used these measurements to calculate body mass index (BMI) as kg/m^2^.

### Assessment of the outcome

The NHANES III cohort study’s mortality information was identified through linkage to the National Death Index through 31 December 2015. The mortality data included the underlying cause of death. For total mortality, we included deaths from all causes. For cancer-specific mortality, we included deaths from malignant neoplasms coded from C00–C97 in the International Classification of Diseases, 10th Edition, Clinical Modification System codes (ICD, 2021) [21, 26, 28]. The follow-up time was determined based on the interval from the 24-h recall interview to the date of death or to 31 December 2015, for those that were censored.

### Statistical analysis

**Fig. 1:**
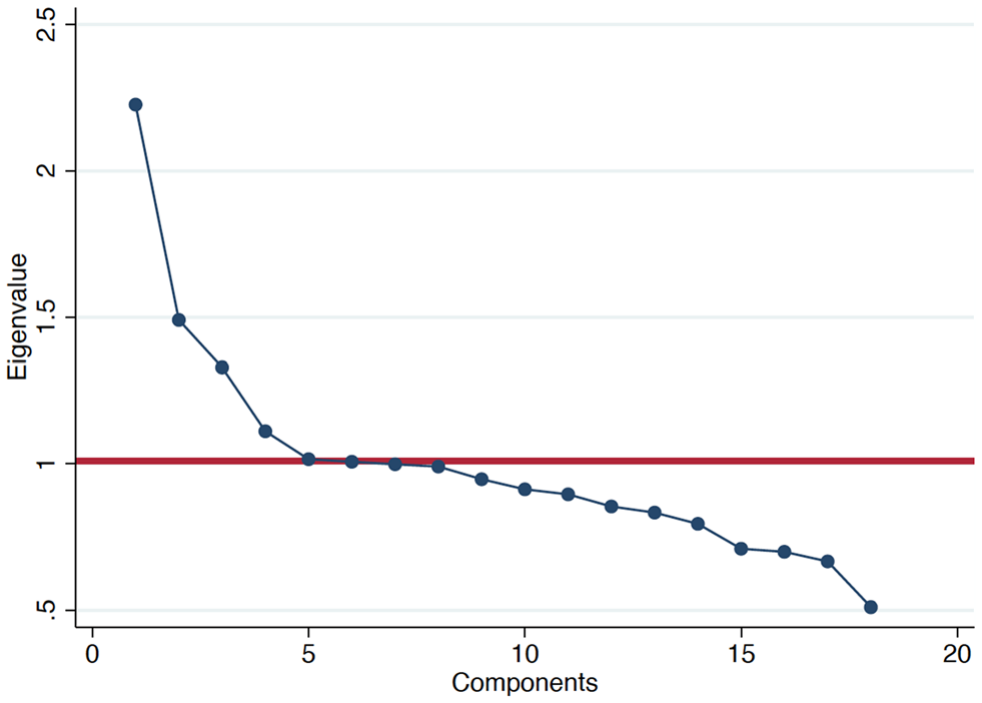
Scree plot used for identification of dietary patterns (components) by principal component analysis. Components with eigenvalues greater than one are considered major dietary pattern scores

We used principal component analysis (PCA) to assess the major dietary patterns in the NHANES III cohort. We tested the sample adequacy for PCA using the Kaiser–Meyer–Olkin (KMO) test. We identified the major dietary patterns based on the components with eigenvalues greater than one and a confirmatory scree test (Fig. 1). Then, we calculated scores for each of the dietary patterns. Food groups with absolute scoring coefficients > 0.3 were considered substantial contributors to a pattern. We evaluated the distribution of population characteristics by quintiles of dietary pattern scores and tested for trend analysis. We used 1-way ANOVA for quantitative variables and Chi-square tests for qualitative variables to identify significant differences across quintile of dietary pattern scores.

After confirming the proportionality of hazards assumption, we used Cox regression models with the length of follow-up as the primary time variable to test the association between dietary pattern adherence and mortality due to all causes and mortality due to cancer. Hazard ratios (HR) with 95% confidence intervals (CI) across quintiles of dietary patterns were calculated using the lowest quintile of adherence as the reference category. In the multivariable models, potential confounders included as covariates were age, sex, total energy intake, smoking status, baseline BMI and previous history of chronic diseases (diabetes, hypertension, stroke, heart attack) and cancer. Also, tests of linear trend across successive quintiles of adherence to each pattern were conducted, treating the variable as continuous. We considered *p* values < 0.05 statistically significant. Sample weights were used to calculate population estimates to account for unequal probability of selection. All analyses were performed using STATA version 13.1.

## Results

### Dietary pattern assessment

We identified four major dietary patterns, explaining 33% of the total variance in the food groups’ consumption in the study population. The factor loadings are shown in Table 2. The first dietary pattern was named ‘prudent’ because it had a high score for vegetables, fruits, and a low score in meat and oils. The second dietary pattern was named ‘western’ since it was characterized by a high score for red meat, sweets, pastries, oils and low scores for vegetables and fruits. The third pattern was named ‘traditional’ because it had high scores in traditional foods, including red meat, eggs, legumes, potatoes, and bread. Finally, the fourth dietary pattern was labeled as ‘fish and alcohol’ due to the notably high score for alcoholic beverages compared to other foods, fish and seafood.

**Table 2:** Factor loading matrix for the major factors identified by using food consumption data from NHANES III^a^

	‘Prudent’ pattern	‘Western’ pattern	‘Traditional’ pattern	‘Fish and alcohol’ pattern
Dairy	0.01	0.03	0.06	−0.11
Red meat	−0.21	**0.41**	**0.36**	0.14
Poultry	0.21	−0.20	−0.09	0.26
Processed meat	0.04	0.03	0.02	0.03
Fish	0.21	−0.26	0.11	**0.45**
Eggs	0.21	0.29	0.06	−0.18
Vegetables	**0.43**	−**0.30**	−0.02	0.07
Fruit	**0.33**	−**0.34**	0.21	−0.12
Legumes	0.25	−0.22	**0.53**	−0.01
Potatoes	**0.33**	0.22	**0.39**	0.02
Nuts and seeds	0.24	0.12	0.28	−0.15
Cereal	0.21	−0.15	−0.25	−**0.43**
Bread	**0.35**	0.17	**0.30**	−0.22
Sweets and pastries	0.14	**0.46**	0.11	0.19
Caffeinated beverages	0.02	0.06	−0.01	−0.06
Soda	−0.21	0.09	0.16	0.28
Alcohol beverages	0.05	0.15	0.18	**0.42**
Oils	0.27	**0.37**	0.21	−0.03

^a^Bolded numbers correspond to absolute values > 0.30 and are considered significant contributors to the pattern

‘Starch’ pattern explained 12% of the variance; the ‘refined sugars & high protein’ pattern explained 8%; the ‘traditional’ pattern 7%; and the ‘fish and alcohol’ pattern explained 6% of the variance

### Demographic characteristics

The mean age of the 13,446 participants was 46.89 years. The mean follow-up of participants was 19.59 years, with a minimum and maximum follow-up of 0.10 and 27.2 years, respectively. During this period, 4,963 deaths were registered. The leading causes of death were cardiovascular disease (23.65%), cancer (21.89%), cerebrovascular disease (6.64%), and other reasons (47.8%). The mean age of death for the deceased participants was 64.10 years. In Table 3, the baseline characteristics of the participants are shown across the quintiles for the dietary patterns. On average, the participants with the highest adherence to the ‘prudent’ dietary pattern were more likely to be men, older, had more years of education, and were less likely to be current smokers. They were also less likely to have a previous history of stroke and cancer; they were leaner despite having a higher calorie intake; and they consumed higher amounts of fiber, carbohydrates, and protein and consumed a lower amount of fats and alcohol.

**Table 3:** Baseline characteristics and nutrient consumption of the study population by quintiles of dietary patterns (*n* = 13,465)

	‘Prudent’ pattern		‘Western’ pattern		‘Traditional’ pattern		‘Fish and alcohol’ pattern	
	*Q1*	*Q5*	*Q1*	*Q5*	*Q1*	*Q5*	*Q1*	*Q5*
*n*	2,694	2,693	2,694	2,693	2,694	2,693	2,694	2,693
	%							
*Sex, Male*	8.04	8.73*	8.55	9.92*	11.08*	7.06	8.25	8.31
Current smoker	5.57*	3.97	3.01	6.92*	2.97	5.86*	3.49	5.76*
Ischemic Heart Disease	0.81	0.99	1.02	0.65	0.61	1.41	1.31	0.51
Stroke	0.52*	0.43	0.47	0.78	0.83*	0.37	1.07	0.25
Hypertension	5.40	5.25	6.59	4.09*	6.23*	4.60	5.23	4.85
Diabetes	9.77	9.26	9.57	9.37	9.28*	8.13	8.49	8.79
Cancer	1.67*	1.07	1.25	1.48*	0.52	2.70	1.93	1.62
	*Mean ± Standard deviation*							
Age*, years*	42.45 ± 18.16	49.l5 ± 20.35*	50.27 ± 18.74	41.83 ± 18.92*	56.44 ± 20.28*	41.56 ± 17.31	55.24 ± 21.64*	43.09 ± 16.95
BMI*,* kg/m^2^	26.83 ± 5.62*	25.22 ± 5.11	26.56 ± 5.09	26.97 ± 5.52^*^	26.64 ± 5.23*	25.74 ± 5.01	25.94 ± 4.92	26.17 ± 5.49
Education*, years*	11.34 ± 3.32*	10.91 ± 3.97	11.06 ± 4.30	10.38 ± 2.80*	9.02 ± 4.24	12.15 ± 3.20*	10.09 ± 3.92	12.42 ± 3.18*
Total energy intake (kcal)	1886.67 ± 780.93	2087.31 ± 812.13*	1823.65 ± 737.76	2222.53 ± 823.28*	2111.32 ± 817.57*	1900.55 ± 757.18	1902.83 ± 775.54	2051.16 ± 793.49*
kcal from fat	34.06 ± 9.46*	32.84 ± 9.02	30.03 ± 9.53	35.46 ± 8.61*	32.69 ± 8.98	32.70 ± 9.27	31.95 ± 8.98	33.77 ± 9.56*
kcal from saturated fat	11.27 ± 3.99*	10.61 ± 3.90	9.60 ± 3.92	12.10 ± 3.82*	10.76 ± 3.93	10.83 ± 3.90	11.17 ± 3.95	10.98 ± 3.93
kcal from monounsaturated fat	12.91 ± 4.02*	12.34 ± 3.96	11.04 ± 4.10	13.58 ± 3.73*	12.21 ± 3.87	12.28 ± 4.12	12.40 ± 3.89	12.67 ± 4.13*
kcal from polyunsaturated fat	7.22 ± 3.68	7.22 ± 3.31	6.88 ± 3.57	7.06 ± 3.21*	6.98 ± 3.41	7.06 ± 3.39	6.73 ± 3.11	7.47 ± 3.72*
kcal from protein	15.12 ± 4.96	15.95 ± 4.98*	16.74 ± 5.16*	14.57 ± 4.61	16.04 ± 4.78*	15.58 ± 4.56	14.67 ± 4.43	15.75 ± 5.23*
kcal from carbohydrates	49.32 ± 11.64	50.82 ± 10.79*	52.21 ± 11.32*	48.30 ± 10.83	50.54 ± 10.93	50.35 ± 11.10	51.13 ± 10.79	47.41 ± 11.52
kcal from alcohol	1.50 ± 1.02*	0.39 ± 0.2	1.02 ± 0.53	1.67 ± 0.83*	0.73 ± 0.45	1.37 ± 0.24*	2.25 ± 0.78	3.07 ± 0.89*
Dietary fiber (g)	13.36 ± 8.95	18.68 ± 11.38*	15.16 ± 10.89*	14.32 ± 8.82	17.38 ± 12.42	16.94 ± 9.62	16.28 ± 11.02	15.20 ± 9.27

Q1: quintile 1, lowest consumption; Q5: quintile 5, highest consumption

*p < 0.05 based on analysis of variance or Chi-square test

The participants with the highest adherence to the ‘western’ dietary pattern were more likely to be male, younger, current smokers, have a higher BMI, and have fewer years of education. Additionally, they were more likely to report a history of hypertension and cancer. On average, they had a higher caloric intake, higher consumption of total fat and alcohol, and lower protein consumption, carbohydrates, and dietary fiber.

Those with the highest consumption of the ‘traditional’ and the ‘fish and alcohol’ dietary patterns were more likely to be men, younger, current smokers, and have more years of education. Nonetheless, the participants with the highest adherence to the ‘traditional’ diet were less likely to have a previous history of chronic diseases, such as stroke, hypertension, and diabetes at baseline, probably due to their age. Those with the consumption of the ‘traditional’ pattern consumed less protein and more alcohol. And those with the highest consumption of the ‘fish and alcohol’ pattern had higher calorie intake and consumed more fats, protein, and alcohol.

### Dietary patterns and total mortality

There were 4,963 deaths due to all causes among the 13,446 participants from NHANES III cohort. The results of the study showed that the lowest risk of total mortality was found among those with the highest adherence to the ‘prudent’ dietary pattern compared to those with the lowest (5th vs. 1st quintile HR 0.86, 95% CI 0.78–0.94) in the age-adjusted model showing that the ‘prudent’ diet reduced the risk of total mortality over the observation period. In the fully adjusted model, the inverse association between adherence to the ‘prudent’ pattern became attenuated but remained significant (5th vs. 1st quintile HR 0.90, 95% CI 0.82–0.98). Furthermore, there was evidence of a significant trend in both models (*p* for trend 004; and *p* for trend 0.007, respectively). Conversely, those in the highest group of consumption of the ‘western’ (5th vs. 1st quintile HR 1.38, 95% CI 1.26–1.51) and the ‘traditional’ (5th vs. 1st quintile HR 1.32, 95% CI 1.21–1.44) dietary patterns had the highest risk of total mortality in the age-adjusted models. In the fully adjusted models, the positive associations between greater adherence to the ‘western’ (5th vs. 1st quintile HR 1.22, 95% CI 1.11–1.34) and the ‘traditional’ (5th vs. 1st quintile HR 1.16, 95% CI 1.06–1.27) dietary patterns and total mortality became attenuated; nonetheless, they remained significant and showed a positive trend. In this study, the association between the ‘fish and alcohol’ dietary pattern and total mortality in the age-adjusted and the fully adjusted models was marginally significant for those in the highest consumption group (5th vs. 1st quintile HR 0.94, 95% CI 0.87–1.01; and 5th vs. 1st quintile HR 0.96, 95% CI 0.89–1.03, respectively). The HR and 95% CI for total mortality according to baseline adherence to the four dietary patterns are shown in Table 4.

**Table 4:** Hazard ratios for total mortality according to quintiles of adherence categories of dietary patterns in NHANES III

	Quintiles of dietary pattern consumption					
	*Q1*	*Q2*	*Q3*	*Q4*	*Q5*	*p for trend*
*‘Prudent’ pattern*						
All-cause of death, N (%)	825 (16.62)	949 (19.12)	1,006 (20.27)	1,090 (21.96)	1,093 (22.02)	
Person-years	55,307.99	53,588.89	52,618.99	51,385.39	50,934.99	
Age-adjusted model	1	0.90 (0.82–0.99)	0.90 (0.81–0.98)	0.89 (0.81–0 .98)	0.86 (0.78–0.94)	0.004
Fully adjusted model	1	0.96 (0.88–1.06)	0.94 (0.89–0.99)	0.91 (0.83–0 .99)	0.90 (0.82–0.98)	0.007
*‘Western’ pattern*						
All-cause of death, N (%)	1,047 (21.10)	1,069 (21.54)	1,037 (20.89)	961 (19.36)	849 (17.11)	
Person-years	52,029.39	51,462.89	52,213.09	53,409.99	54,720.89	
Age-adjusted model	1	1.11 (1.02–1.21)	1.15 (1.06–1.25)	1.21 (1.11–1.32)	1.38 (1.26–1.51)	< 0.001
Fully adjusted model	1	1.07 (0.98–1.17)	1.10 (1.01–1.19)	1.12 (1.02–1.22)	1.22 (1.11–1.34)	0.020
*‘Traditional’ pattern*						
All-cause of death, *n* (%)	806 (16.24)	783 (15.78)	909 (18.32)	1,028 (20.71)	1,437 (28.95)	
Person-years	55,049.09	55,459.09	54,048.29	52,586.49	46,693.29	
Age-adjusted model	1	1.03 (0.95 1.12)	1.03 (0.95–1.13)	1.15 (1.05–1.29)	1.32 (1.21–1.44)	< 0.001
Fully adjusted model	1	0.99 (0.91–1.07)	0.94 (0.86–1.02)	1.04 (0.95–1.14)	1.16 (1.06 1.27)	0.011
*‘Fish and alcohol’ pattern*						
All-cause of death, *n* (%)	1,491(30.04)	1,120 (22.57)	886 (17.85)	733 (14.77)	733 (14.77)	
Person-years	44,877.99	51,143.39	54,655.29	56,900.79	56,258.79	
Age-adjusted model	1	0.99 (0.92–1.06)	0.95 (0.88–1.02)	0.96 (0.89–1.03)	0.94 (0.87–1.01)	0.010
Fully adjusted model	1	0.92 (0.85–0.99)	0.89 (0.78–1.01)	0.93 (0.86–1.00)	0.96 (0.89–1.03)	0.037

Fully adjusted model: Adjusted for age, sex, energy intake, smoking status, BMI and chronic diseases (diabetes, stroke, hypertension, ischemic heart disease, and cancer)

### Dietary patterns and mortality due to cancer

In this study, there were 1,077 cancer deaths over the study period. Participants with the highest consumption of the ‘western’ pattern had the highest risk of cancer mortality in the age-adjusted model (5th vs. 1st quintile HR 1.34, 95% CI 1.16–1.52). In the fully adjusted model, the positive association between the highest adherence to the ‘western’ pattern and cancer mortality remained significant after accounting for demographic characteristics and comorbidities (5th vs. 1st quintile HR 1.33, 95% CI 1.10–1.62). Similar results were found among those with the highest consumption to the ‘traditional’ pattern in age-adjusted model (5th vs. 1st quintile HR 1.17, 95% CI 1.06–1.28), and the fully adjusted model (5th vs. 1st quintile HR 1.15, 95% CI 1.06–1.24). The associations between adherence to the ‘prudent’ or the ‘fish and alcohol’ pattern and mortality due to cancer in the age-adjusted and fully adjusted models were not statistically significant. The HR and 95% CI for mortality due to cancer according to baseline adherence to the four dietary patterns are shown in Table 5.

**Table 5:** Hazard ratios for mortality due to cancer according to quintiles of adherence categories of dietary patterns in NHANES III

	Quintiles of dietary pattern consumption					
	*Q1*	*Q2*	*Q3*	*Q4*	*Q5*	*p for trend*
*‘Prudent’ pattern*						
Death due to cancer, N (%)	169 (15.69)	213 (19.78)	213 (19.78)	241 (22.38)	241 (22.38)	
Person-years	55,307.99	53,588.89	52,618.99	51,385.39	50,934.99	
Age-adjusted model	1	1.03 (0.84–1.27)	1.01 (0.82–1.24)	1.00 (0.82–1.22)	1.04 (0.85–1.27)	0.854
Fully adjusted model	1	1.08 (0.88–1.32)	1.05 (0.86–1.29)	1.02 (0.84–1.25)	1.07 (0.87–1.30)	0.919
*‘Western’ pattern*						
Death due to cancer, N (%)	218 (20.24)	213 (19.68)	225 (20.89)	241 (18.29)	225 (20.89)	
Person-years	52,029.39	51,462.89	52,213.09	53,409.99	54,720.89	
Age-adjusted model	1	1.05 (0.87–1.27)	1.11 (1.08–1.15)	1.16 (0.05–1.27)	1.34 (1.16–1.52)	< 0.001
Fully adjusted model	1	1.06 (0.88–1.28)	1.02 (0.84–1.23)	1.17 (0.06–1.28)	1.33 (1.10–1.62)	0.789
*‘Traditional’ pattern*						
Death due to cancer, *n* (%)	196 (18.20)	213 (15.41)	211 (19.59)	241 (21.73)	270 (25.07)	
Person-years	55,049.09	55,459.09	54,048.29	52,586.49	46,693.29	
Age-adjusted model	1	0.98 (0.81–1.19)	0.99 (0.81–1.21)	1.15 (1.05–1.20)	1.17 (0.06–1.28)	0.012
Fully adjusted model	1	1.01 (0.83–1.20)	0.97 (0.80–1.18)	1.14 (1.05–1.19)	1.15 (1.06–1.24)	0.829
*‘Fish and alcohol’ pattern*						
Death due to cancer, *n* (%)	301 (27.95)	213 (20.71)	201 (18.66)	241 (15.32)	187 (17.36)	
Person-years	44,877.99	51,143.39	54,655.29	56,900.79	56,258.79	
Age-adjusted model	1	0.88 (0.74–1.05)	0.88 (0.73–1.05)	0.86 (0.72–1.00)	0.89 (0.74 1.07)	0.015
Fully adjusted model	1	0.86 (0.72–1.02)	0.84 (0.68–1.00)	0.80 (0.61–0.99)	0.93 (0.80–1.08)	0.627

Fully adjusted model: Adjusted for age, sex, energy intake, smoking status, BMI and chronic diseases (diabetes, stroke, hypertension, ischemic heart disease, and cancer)

## Discussion

Most studies in the United States have focused on investigating associations between specific dietary factors and chronic diseases [12–14].The association between the adherence dietary patterns and total mortality and mortality due to cancer in the general population is still limited. Dietary patterns condense information about food consumption, reflect diet composition, and capture the overall effect of dietary exposures on health and disease. This study evaluated the association between dietary patterns, total mortality, and cancer mortality in the NHANES III adult cohort in the U.S. Four previously described dietary patterns were found in this study at baseline. The ‘prudent’ dietary pattern, with high intakes of vegetables, fish, fruits, and legumes [29–33]; the ‘western’ dietary pattern, rich in sweets and oils, and low in fruits and vegetables [29–33]; the ‘traditional’ dietary pattern, rich in bread, legumes, eggs; [34, 35]; and the dietary pattern characterized by high consumption of alcoholic beverages [36, 37]. On average, those with the highest consumption of the ‘prudent’ dietary pattern were more likely to adhere to healthier behaviors and less likely to report a previous history of chronic diseases and cancer, in contrast with the participants with the highest consumption of the other patterns. Previous studies support this evidence and demonstrate a correlation between dietary behaviors and lifestyle choices [38, 39].

Our findings confirmed the hypothesis that dietary patterns characterized by consuming nutrient-rich foods are associated with lower total mortality, and dietary patterns that reflect poor nutritional habits are associated with higher total mortality. Herein, the lowest risk of total mortality was among those with the highest adherence to the ‘prudent’ dietary pattern. The ‘prudent’ pattern is characterized by consuming nutrient-rich foods high in antioxidants and vitamins. Prudent diets contain a complex combination of antioxidant and prooxidant elements that can modify the human body’s oxidative status. The oxidative stress resulting from imbalances between reactive oxygen species and the antioxidant defense is a common factor in many pathological conditions [40]. Oxidative stress has been related to cardiovascular disease, cancer, and other chronic diseases that account for a significant portion of deaths in the United States [41].

In this study, participants with the highest consumption of the ‘western’ dietary pattern showed the highest risk of total mortality. The ‘western’ dietary pattern with high consumption of sweets and oils and low consumption of vegetables and fruits was associated with an increased risk of chronic diseases and cancer, probably due to micronutrient deficiencies that lead to several pathophysiological events increase the risk of chronic disease. Westernized diets are known to be deficient in magnesium, zinc, folate, and vitamins C, E, and K [42, 43]. Deficiencies in the micronutrients above were previously related to metabolic syndrome [44], cardiovascular disease [45, 46], and coronary heart disease [47, 48], which are principal causes of mortality in the U.S.

Our findings did not show an association between the ‘traditional’ or the ‘fish and alcohol’ pattern and total mortality. The ‘traditional’ dietary pattern has not been extensively studied in the United States, where traditional foods, inherited from the native communities, served as the foundation for the contemporary diet. Nonetheless, acculturation led to increasing consumption of a westernized diet in native communities, increasing chronic diseases [49]; therefore, more research is needed to understand the factors that influence the adherence to the ‘traditional’ diet and its effects on health and mortality. Alcohol consumption has been previously linked with increased mortality; nonetheless, the harmful effects depend on the level of alcohol consumption and the amount of time that individuals consume alcoholic beverages [50, 51]. Furthermore, these effects depend on individuals’ characteristics, such as their age and cardiovascular health [52, 53]. In line with these findings, our study showed that individuals with the highest adherence to the alcohol pattern were younger and less likely to have a previous history of chronic diseases and cancer. Moreover, the harmful effects of alcohol consumption, for those with the highest adherence to the ‘fish and alcohol’ dietary pattern, may have been compensated by the high intake of fish and seafood, which has been linked with positive health outcomes such as reduced cardiovascular risk [54], and all-cause mortality [55].

We partially confirmed our second hypothesis and provided evidence that dietary patterns that reflect lower quality nutrition are associated with higher cancer mortality. Our findings suggest that participants with the highest consumption of the ‘western’ and the ‘traditional’ dietary pattern had the highest risk of mortality due to cancer, probably due to the high consumption of fats. Carrol and colleagues reviewed experimental and epidemiological studies on the role of dietary fat and cancer. Their findings suggested that high‐fat diets increase cancer risk, and total dietary fat correlates with cancer mortality [56]. Similar findings were found across different types of cancers [57, 58]. This study found no association between adherence to the ‘prudent’ or the ‘fish and alcohol’ pattern and cancer mortality. Diets rich in fruits and vegetables are widely considered beneficial to health since they contain antioxidants, vitamins, and dietary fiber responsible for the benefits.

Nonetheless, the results of several studies have been inconsistent in the association between the consumption of fruits and vegetables and cancer risk. Additionally, a recent meta-analysis indicated that higher consumption of fruits and vegetables was not significantly associated with cancer mortality risk [59]. The evidence presented herein suggests that efforts to increase consumption of a ‘prudent’ dietary pattern may reduce the risk of total mortality, and the benefit for cancer mortality remains possible.

### Strengths and limitations

This study’s key strengths include the long length of follow-up time and the large nationally representative sample of adults in the United States that were included. Furthermore, it is the first study investigating the role of adherence to dietary patterns and total mortality, and cancer mortality in the United States, to the best of our knowledge. Nonetheless, the study has limitations. First, the study used self-reported data that could be subject to recall bias due to the lack of medical records validation to confirm the medical diagnoses. Second, residual confounding may also be a limitation due to unmeasured socioeconomic variables. Finally, the study analyzed baseline dietary information and did not capture information about diet changes over time.

## Conclusion

In this nationally representative cohort, adherence to the ‘western’ and the ‘traditional’ dietary pattern was strongly associated with an increased risk of total mortality and mortality due to cancer. The ‘prudent’ dietary pattern was associated with a decreased risk of total mortality, but not due to cancer. The evidence presented herein suggests that efforts to increase consumption of a ‘prudent’ dietary pattern may reduce the risk of total mortality, and the benefit for cancer mortality remains possible.

## Data Availability

Not applicable.
